# The Experimental Registration of the Evanescent Acoustic Wave in YX LiNbO_3_ Plate

**DOI:** 10.3390/s21062238

**Published:** 2021-03-23

**Authors:** Andrey Smirnov, Boris Zaitsev, Andrey Teplykh, Ilya Nedospasov, Egor Golovanov, Zheng-hua Qian, Bin Wang, Iren Kuznetsova

**Affiliations:** 1 Kotelnikov Institute of Radio Engineering and Electronics of RAS, 125009 Moscow, Russia; ianedospasov@mail.ru (I.N.); kasper_96.96@mail.ru (E.G.); kuziren@yandex.ru (I.K.); 2 Kotelnikov Institute of Radio Engineering and Electronics of RAS, Saratov Branch, 410019 Saratov, Russia; zai-boris@yandex.ru (B.Z.); teplykhaa@mail.ru (A.T.); 3 State Key Laboratory of Mechanics and Control of Mechanical Structures, College of Aerospace Engineering, Nanjing University of Aeronautic and Astronautic, Nanjing 210016, China; qianzh@nuaa.edu.cn (Z.-h.Q.); wangbin1982@nuaa.edu.cn (B.W.)

**Keywords:** evanescent waves, backward waves, lithium niobate plates, interdigital transducers, zero group velocity point

## Abstract

Evanescent acoustic waves are characterized by purely imaginary or complex wavenumbers. Earlier, in 2019 by using a three dimensional (3D) finite element method (FEM) the possibility of the excitation and registration of such waves in the piezoelectric plates was theoretically shown. In this paper the set of the acoustically isolated interdigital transducers (IDTs) with the different spatial periods for excitation and registration of the evanescent acoustic wave in Y-cut X-propagation direction of lithium niobate (LiNbO_3_) plate was specifically calculated and produced. As a result, the possibility to excite and register the evanescent acoustic wave in the piezoelectric plates was experimentally proved for the first time. The evanescent nature of the registered wave has been established. The theoretical results turned out to be in a good agreement with the experimental ones. The influence of an infinitely thin layer with arbitrary conductivity placed on a plate surface was also investigated. It has been shown that the frequency region of an evanescent acoustic wave existence is very sensitive to the changes of the electrical boundary conditions. The results obtained may be used for the development of the method of the analysis of thin films electric properties based on the study of evanescent waves.

## 1. Introduction

Recently, the interest of researchers has been attracted to the so-called evanescent acoustic waves that exist in confined media. These waves, in contrast to acoustic Lamb waves and waves with shear horizontal polarization, are characterized by a purely imaginary or complex wave number [1,2,3]. In the case of a purely imaginary wavenumber or with a significant excess of the imaginary part relative to the real part, the evanescent mode corresponds to vibration near the source of the external force, which decays exponentially with the distance from the source and does not transfer energy [1,2,3,4]. The theoretical studies of the dispersion dependences of such waves propagated in isotropic plates [1,2,3,4], piezoelectric plates of cubic symmetry [5], functionally graded piezoelectric-piezomagnetic plates [6], phononic crystals [7] were carried out earlier. These studies were carried out for non-dissipative media with zero viscosity. The evanescent waves have also been investigated in viscoelastic anisotropic plates [8,9], cylinders [10], multilayer structures [11,12], and spherically curved plates [13]. It has been shown that waveguide modes with real and complex wave numbers in plates, tubes, cylindrical shells become coupled when immersed in a liquid [11,12,14,15,16,17]. In recent years, studies have been actively carried out on the waves characterized by a complex wave number in various acoustic metamaterials [18,19] and corrugated waveguides [20]. Recently, the possibility of the existence of acoustic spin was theoretically shown [21] and experimentally confirmed [22]. The existence of the transverse spin in evanescent waves with orthogonal real and imaginary parts of the wave number was theoretically demonstrated in [23]. 

It should be noted that the problem of excitation of the evanescent acoustic waves is interesting from both a fundamental and a practical point of view. Earlier it has been shown that when Lamb waves are reflected from the edge of the plate, not only propagating waves are excited, but also evanescent waves that exist only near the edge of the plate [1,24,25,26,27,28,29,30]. The existence of evanescent Lamb waves has been experimentally proven at free-edge boundaries [31,32], in phononic crystals [7,19], elastic metamaterial [18] and the possibility of their using for non-destructive control has been confirmed in [33,34,35,36]. These waves also could be used for development of a planar acoustic transducer for near field acoustic communication [37], passive pressure sensors for harsh-environment applications [38], new air-coupled ultrasonic for non-destructive techniques [39] and in acoustofluidic chips for microscale manipulation [40]. Despite these studies, there is still a need to develop methods for the excitation and registration of these waves. Recently for the excitation and registration of backward acoustic waves in piezoelectric plates a method based on the use of a set of interdigital transducers (IDTs) with different periods was proposed in [41]. Later the possibility of using this method for excitation and registration of evanescent acoustic waves in lithium niobate and potassium niobate plates was theoretically shown in [42]. However, for realization of real nondestructive analysis technique it is necessary to confirm experimentally the reliability of the method proposed earlier. Another possible field of application of these waves is the development of controlled acoustoelectronic devices. One of the control methods is the arrangement of a heterostructure with a conductivity variable by an applied electric field on the surface of the plate [43,44]. Similar studies were carried out earlier for forward and backward acoustic waves in various piezoelectric materials [45,46,47,48,49]. As for evanescent acoustic waves in piezoelectric plates, such works are currently absent.

So, in this paper the possibility of registration of the evanescent acoustic waves in a piezoelectric plate by using a system of the acoustically isolated IDTs with different spatial periods was firstly experimentally shown. The experiments confirmed the existence of an evanescent backward wave in Y-X LiNbO_3_ plate. As well the influence of an infinitely thin layer with arbitrary surface conductivity has been firstly investigated. It has been shown that the frequency region of an evanescent acoustic wave existence is very sensitive to the changes in the electrical boundary conditions. The calculations have shown that an increase in the layer conductance can reduce the attenuation of an evanescent wave down to zero and thereby transform this wave into a propagating one. 

## 2. Materials and Methods

### 2.1. Theoretical Methods

#### 2.1.1. Boundary Transfer Matrix Method

Earlier it has been found that a backward antisymmetric acoustic wave of the 1st order (A_1_) exists in Y-cut of lithium niobate (LiNbO_3_) plate for X-propagation direction [41]. Later it has been theoretically shown that in the region near a zero group velocity (ZGV) point of the aforementioned A_1_ wave the evanescent backward acoustic wave also exists [42]. 

For the first step of this study the phase velocity, mechanical displacements and electrical potential distribution of an A_1_ wave in YX LiNbO_3_ plate with the thickness of 490 µm were calculated by using the matrix method [50]. This method allows one to consider the wave propagation far from source of excitation.

**Figure 1:**
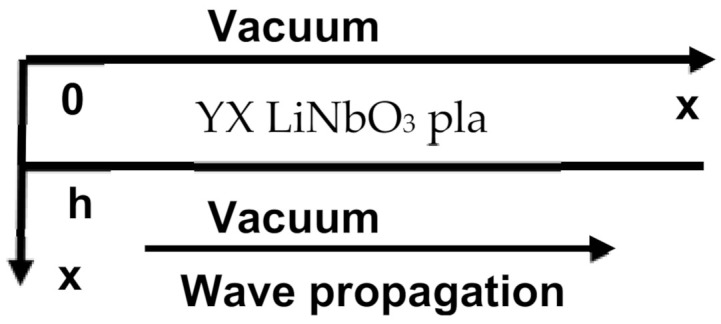
Geometry of the problem.

The geometry of the problem is presented in Figure 1. Standard motion equation and Laplace’s Equation (1), and constitutive equations for piezoelectric medium (2) were used for calculation [51,52].

(1) $$\rho \partial^{2}U_{i}/\partial t^{2}=\partial T_{ij}/\partial x_{j},\;\partial D_{j}/\partial x_{j}=0,$$

(2) $$T_{ij}=C_{ijkl}^{}\partial U_{l}/\partial x_{k}+e_{kij}^{}\partial \Phi /\partial x_{k},\;D_{j}=-\varepsilon_{jk}^{}\partial \Phi /\partial x_{k}+e_{jlk}^{}\partial U_{l}/\partial x_{k}.$$

Here, *E_i_* and *U_i_* are the components of the electric field intensity and mechanical particle displacement. *t* and *x_j_* are the time and coordinate. *T_i_*_j_ is the component of mechanical stress tensor. *D_j_* is the component of electric displacement. Φ and *ρ* are the electric potential and density. *C_ijkl_*, *e_ikl_* and *ε_jk_* are the elastic, piezoelectric and dielectric constants of a piezoelectric material, respectively. We also used the Laplace’s Equation (3) for vacuum [51,52]:

(3) $$\partial D_{j}^{v_{1}}/\partial x_{j}=0,\partial D_{j}^{v_{2}}/\partial x_{j}=0,$$

where $D_{j}^{v_{1}}=-\varepsilon_{0}\partial \Phi^{v_{1}}/\partial x_{j}$ and $D_{j}^{v_{2}}=\varepsilon_{0}\partial \Phi^{v_{2}}/\partial x_{j}$. Here, indices *v_1_* and *v_2_* denote quantities relating to vacuum in the planes *x_3_* = 0 and *x_3_* = *h*, respectively, *ε_0_* is the dielectric constant of vacuum.

As boundary conditions we used the continuity of the potential and normal component of electrical displacement, as well equality to zero of the normal components of the mechanical stress tensor for the interfaces vacuum/plate (*x_3_* = 0 and *x_3_* = *h*) [51,52].

(4) $$T_{3j}=0,\Phi^{v_{1}}=\Phi ,\Phi^{v_{2}}=\Phi ,D_{3}^{v_{1}}=D_{3},\;D_{3}^{v_{2}}=D_{3}.$$

For the study of the influence of an infinitely thin layer with arbitrary conductivity placed in the plane *x_3_* = 0 on the properties of an evanescent wave the next electrical boundary conditions were used [46]:

(5) $$\Phi^{v_{1}}=\Phi ;D_{3}^{v_{1}}-D_{3}=\delta .$$

Here, *δ* is the surface charge density that is related to the density of surface current [46].

(6) $$\delta =j\sigma_{S}\Phi^{v_{1}}\omega /V_{ph}^{2}$$

Here, *σ_S_* is the surface conductance of the layer, *j* is the imaginary unit, *ω* = 2*πf* is the angular frequency, *V_ph_* and *f* are the complex phase velocity and frequency of an acoustic wave, respectively.

This problem was solved by the method described in detail in [41,42]. The material constants for LiNbO_3_ were taken from [53].

In order to determine the spatial period of the IDTs or wavelength (*λ*) needed for an experiment the auxiliary lines Re(*V_ph_*) *= λf = (λ/h)(hf)* for different values of *λ* were calculated and plotted. Re(*V_ph_*) means the real part of the acoustic wave phase velocity that has a complex nature in common case. In this case the next formula was used for calculation [1]:

*V_ph_* = (Re(*V_ph_*)^2^ + Im(*V_ph_*)^2^)/Re(*V_ph_*) (7)

#### 2.1.2. FEM Simulation

**Figure 2:**
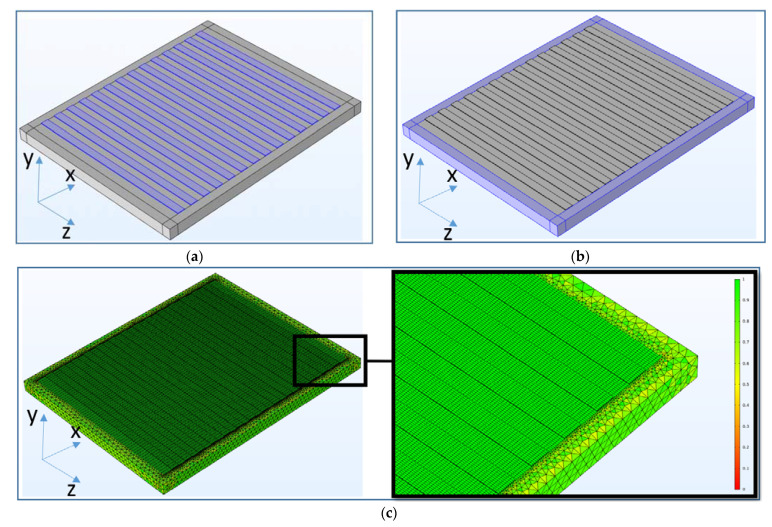
3D Model of the resonator used in the finite element method (FEM) calculation including: (**a**) aluminum electrodes on the surface of the resonator, (**b**) perfectly matching layers, (**c**) meshes used in modeling.

The theoretical analysis described above did not take into account the problem of the excitation of the acoustic waves by an IDT. So, the experimental situation was modeled by using the FEM commercial software COMSOL 5.3. The approach used is described in detail in [41,42]. An image of the resonator model used in the calculations, the location of the perfectly matching layers (PML) and the corresponding mesh are shown in Figure 2.

The PML layer, located on the lateral edges of the plate, prevents the re-reflection of excited waves from the boundaries of the resonator. This leads to the disappearance of false peaks in the simulated resonance curve. In the implementation of these absorbing layers, quadratic damping functions were used. In the region of the plate free of the IDTs, we used mechanical boundary conditions corresponding to the free boundaries, i.e., the mechanical stresses were considered to be equal to zero. In the area of the contact of the IDT’s fingers with the plate, the continuity of mechanical displacements and stresses between the IDT’s fingers and the plate was used as the mechanical boundary conditions. The normal component of the electrical displacement on the plate surface was assumed to be zero. Excitation of an acoustic wave was simulated by periodically changing an electric potential in the contact area of the IDT fingers and a piezoelectric plate. An alternating electric potential was applied to the odd-numbered IDT fingers. The even numbered fingers had zero potential. The metal electrodes were considered as the mass loading. 

The mesh was generated manually during the model compiling. Two types of the meshes (i) for the region of a plate under the electrodes, (ii) for the area of perfectly matching layers were used in the model. In the case (i) for the electrodes and the inter-electrode space the meshes were represented by parallelepipeds. The linear dimensions of the mesh elements in this case correspond to 40 elements per wavelength along the *X*, *Y* and *Z*-axes. 

For the case (ii), an automatically generated tetrahedral mesh was used. The meshing in finite element analysis is an important modeling step. The quality of the generated meshes can be assessed using a special tool of the Multiphysics simulation platform.

Figure 2c shows an image of the mesh, where each mesh element is colored from red to green. The color of the elements indicates their quality. On the right, the graph shows the relative scale of the quality of the elements. The more elements on such graph are closer to 1 in quality (green), the more adequately the model will simulate the real situation. The average quality of the mesh elements is 0.86, which indicates a high accuracy of the simulation results. To avoid erroneous conclusions due to insufficient accuracy and suboptimal mesh selection, the quality of the model was also assessed by a simple iterative method, by successively reducing the size of the mesh elements. In the analysis, the number of elements varied from 4 to 60 per wavelength. It was found that at 20 elements per wavelength, the size of the grid element ceases to affect the form of resonance dependences. In this regard, it was concluded that 40 grid elements that fit along the wavelength are enough to simulate a real experimental situation.

### 2.2. Experimental Study

**Figure 3:**
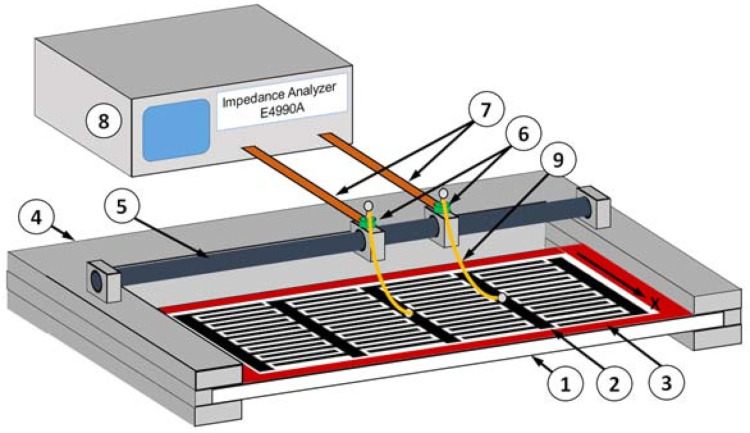
Scheme of the experimental set up: (1) YX LiNbO_3_ plate; (2) system of IDTs; (3) layer of absorbing varnish; (4) support made of textolite; (5) guide shaft; (6) movable dielectric holders; (7) flat contact legs; (8) impedance analyzer; (9) gold wires.

For the experimental study of an evanescent acoustic wave, we used the experimental setup shown in Figure 3. The base was a LiNbO_3_ plate (1) with a thickness of 0.49 mm, on which a set of four IDTs (2) with spatial periods of 1.4, 1.42, 1.44 and 1.46 mm were deposited using photolithography. The width of the each strip in IDT and distance between them was equal to *λ*/4. The normal to the plate and the direction of propagation of the excited waves were oriented along the *Y* and *X* axes, respectively. To prevent reflections of the excited waves from the edges of the plate and ensure the conditions for a traveling wave, the region around the transducers was covered with a layer of absorbing varnish (3) 0.2 mm thick [41]. The lithium niobate plate was fixed in a special support (4) made of textolite. A guide shaft (5) with two movable dielectric holders (6) with flat contact legs (7) was also located on the support. On the one hand, the contact legs provided a reliable connection with the impedance analyzer (8) E4990A (Keysight), and on the other hand, they were connected to the IDT contact areas by means of the gold wires (9) of 25 μm in diameter. These wires were glued in advance to the each contact area of the IDT with conductive glue “Silver Print”. On the other hand, they were soldered to the contact legs using a micro soldering iron.

The measurement technique was as follows. Movable holders with legs were located near the selected IDT, and pre-glued gold wires were soldered to the contact legs. Then the frequency dependences of the real and imaginary parts of the electrical impedance of the selected IDT were measured. Then, gold wires of another transducer were soldered to the contact legs and the measurements were repeated. As a result, the frequency dependences of the real and imaginary parts of the electrical impedance of all four IDTs were obtained.

## 3. Results and Discussion

### 3.1. Theoretical Results

**Figure 4:**
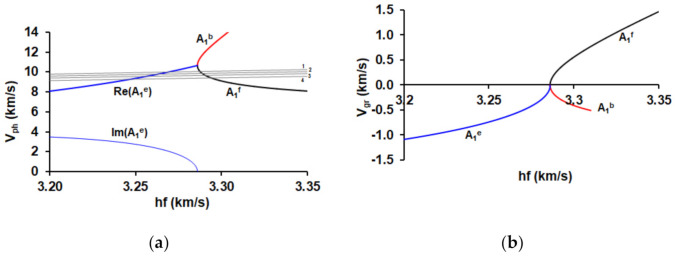
Dependencies of the (**a**) real (Re) and imaginary (Im) parts of the complex phase velocities *V_ph_* and (**b**) the group velocities *V_gr_* on parameter *hf* for the forward (black), backward (red), and evanescent (blue) waves in YX LiNbO_3_ plate. The grey lines are the auxiliary lines for different values of *λ*: 1.46 mm (1), 1.44 mm (2), 1.42 mm (3), and 1.40 mm (4).

As the result of the calculations, carried out by using the approach described in item 2.1.1 the dependencies of the real and imaginary parts of the complex phase velocity *V_ph_* on parameter *hf* (*h* is the plate thickness, *f* is the wave frequency) were plotted (Figure 4a). Three branches A_1_^f^, A_1_^b^ and A_1_^e^ are corresponded to the forward (black), backward (red), and evanescent (blue) wave, respectively. For calculation of the group velocity (*V_gr_*) of the forward and backward waves we used well known formulae by using dispersion curves presented in Figure 4a:

*V_gr_* = d*ω*/d*k*, (8)

where *k* is the real part of the wave number that is much higher than its imaginary part for forward and backward branches. The calculated dependencies *V_gr_* versus parameter *hf* for these branches are presented in Figure 4b.

The evanescent wave is characterized by the complex wave number *k* = Re*k* + *j*Im*k* and complex phase velocity *V_ph_* [42,54] (Figure 4a). By using *V_ph_*(*hf*) dependence we can numerically find the derivative *dV_ph_*/*d(hf*) as follows:

(9) $$\frac{dV_{ph}^{\left(n\right)}}{d{\left(hf\right)}^{n}}=\frac{V_{ph}^{n+1}-V_{ph}^{n}}{hf^{n+1}-hf^{n}}.$$

where the indices *n* and *n* + 1 relate to the values of *V_ph_* and *hf* corresponded to the *n* and *n* + 1 steps of calculation. Then the group velocity at the *n* step can be expressed as follows.

(10) $$V_{gr\_cmplx}^{\left(n\right)}=\frac{\left(hf^{\left(n+1\right)}-hf^{n}\right){\left(V_{ph}^{n}\right)}^{2}}{V_{ph}^{n}hf^{n+1}-V_{ph}^{n+1}hf^{n}}.$$

The final value of *V_gr_* was used in the following form:

(11) Vgr=(ReVgr_cmplx)2+(ImVgr_cmplx)2ReVgr_cmplx.

As mentioned above, the wave number for evanescent waves is complex. In this regard, to calculate the dependence of the real and imaginary parts of the wave number, the following formulas were used:

(12) k=ωReVph+jImVph=ω(ReVph−jImVph)(ReVph)2+(ImVph)2=ωReVph(ReVph)2+(ImVph)2−jωImVph(ReVph)2+(ImVph)2

From here:

(13) Rek=ωReVph(ReVph)2+(ImVph)2;Imk=−jωImVph(ReVph)2+(ImVph)2

The auxiliary lines Re(*V_ph_*) *= λf = (λ/h)(hf)* for different values of *λ* needed for an experiment are presented in Figure 4a by the grey lines.

**Figure 5:**
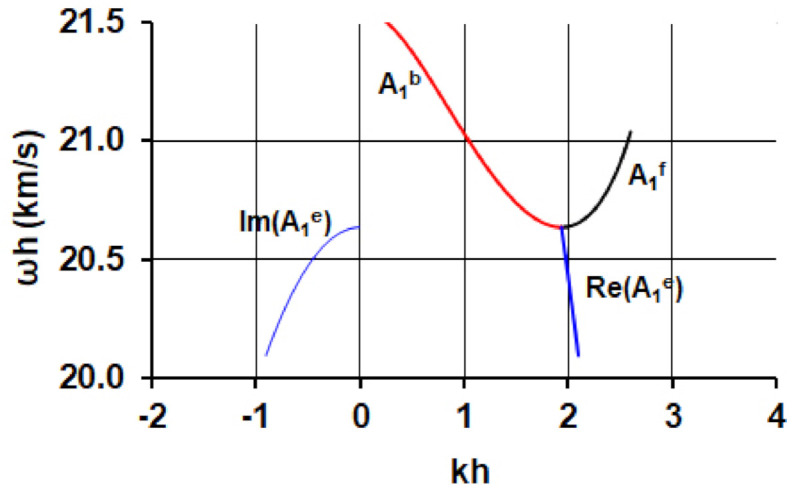
Dispersion curves for real (Re) and imaginary (Im) parts of wave numbers *k* of the forward (black), backward (red), and evanescent waves (blue) in YX LiNbO_3_ plate.

The dispersion curves calculated by using the dependencies presented in Figure 4a are shown in Figure 5.

**Figure 6:**
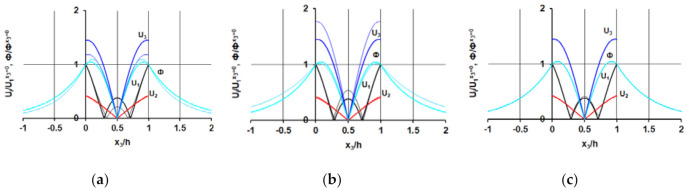
Dependencies of the normalized electric potential value Ф and mechanical displacements *U*_1_, *U*_2_, and *U*_3_ belonging to the forward (**a**), backward (**b**), and evanescent (**c**) waves on the structure thickness *x*_3_/*h* in the regions near (thick lines) and far (thin lines) from ZGV point.

The dependencies of the electric potential value Φ normalized to the surface value Φ^*x*3=0^ and mechanical displacements *U*_1_, *U*_2_, and *U*_3_ normalized to the surface value *U*_1_^*x*3=0^ belonging to the waves under study in the regions near (*hf* = 3286.04 m/s for all branches) and far (*hf* = 3307, 3307 and 3273 m/s for forward, backward and evanescent branches, respectively) from the zero group velocity (ZGV) point are presented in Figure 6.

One can see that, in contrast to the forward and backward waves, the structure of the evanescent wave does not depend on the frequency region of its existence.

**Figure 7:**
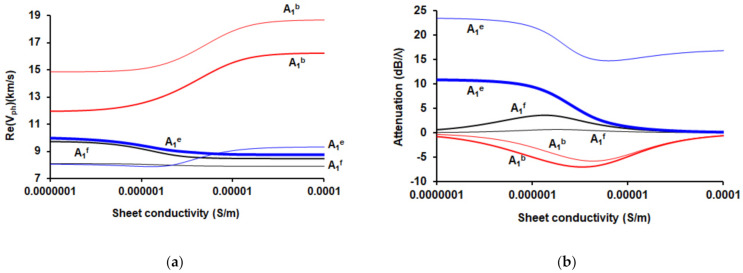
Dependencies of (**a**) the real parts of the phase velocities *V_ph_* and (**b**) attenuation per wavelength on the sheet conductivity of the infinitely thin layer placed in plane *x*_3_ = 0 for the forward (black), backward (red), and evanescent (blue) waves in Y-X LiNbO_3_ plate in the regions near (thick lines) and far (thin lines) from ZGV point.

The dependencies of the real part of the complex phase velocities (Figure 7a) and attenuation per wavelength (Figure 7b) on sheet conductivity for waves under study in the regions near (thick lines) and far (thin lines) from ZGV point are presented in Figure 7.

These figures show that near the ZGV point for a forward wave, as expected, an increase in the conductivity of the layer on the surface of the plate leads to a decrease in its velocity. At the same time, the attenuation increases, reaches a maximum, and decreases. With a rise of the distance from the ZGV point, the dependence of the parameters of the forward wave on the surface conductivity practically disappears. This is associated with a decrease in the coefficient of an electromechanical coupling of this wave with an increase in the parameter *hf*.

For a backward wave, the above dependencies behave in the opposite way, and the attenuation is a negative value. This is due to the opposite direction of the phase and group velocity vectors.

As for the evanescent wave, one can see that near the ZGV point its phase velocity decreases with increasing conductivity, similarly to the forward wave. It can be concluded that the coefficient of an electromechanical coupling of the evanescent wave near the ZGV point is close to the value corresponding to the forward wave. In this case, the attenuation of the evanescent wave decreases with increasing conductivity to zero near the ZGV point. Analysis of the behavior of this wave far from the ZGV point shows that at a certain value of surface conductivity (about 3 × 10^−6^ S/m) this wave disappears. At the same time, its attenuation with increasing conductivity first decreases, reaches a minimum, and then increases. 

**Figure 8:**
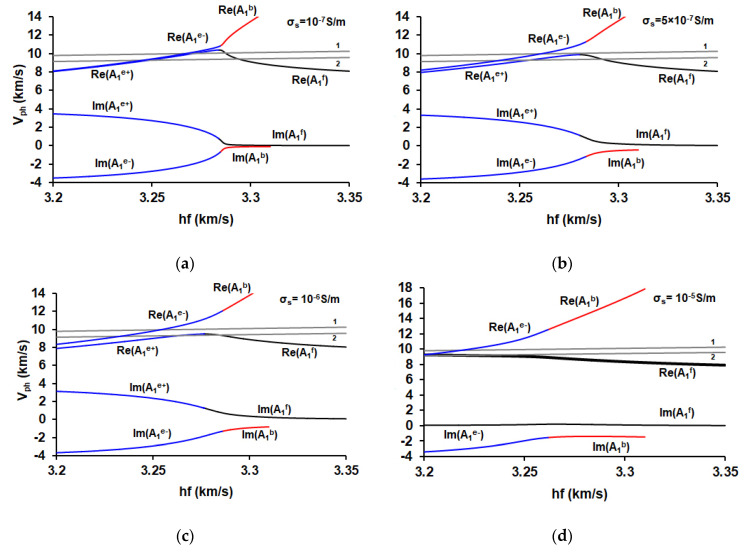
Dependencies of the real and imaginary parts of the phase velocities *V_ph_* of the forward (black), backward (red), and evanescent (blue) waves in Y-X LiNbO_3_ plate on parameter *hf* for various values of sheet conductivity *σ_s_* of the infinitely thin layer placed in plane *x_3_* = 0: (**a**)—10^−7^ S/m, (**b**)—5 × 10^−7^ S/m, (**c**)—10^−6^ S/m, and (**d**)—10^−5^ S/m. The grey lines are the auxiliary lines for different values of *λ*: 1.46 mm (1) and 1.4 mm (2). A_1_^e+^ and A_1_^e−^ correspond to the evanescent wave branches with the positive and negative values of an imaginary part of the complex wave number *k*, respectively.

For a more detailed analysis the dependencies of the real and imaginary parts of the complex phase velocity *V_ph_* of forward, backward and evanescent waves on the parameter *hf* for various values of surface conductivity 10^−7^ S/m, 5 × 10^−7^ S/m, 10^−6^ S/m, and 10^−5^ S/m are shown in Figure 8.

Figure 8 clearly shows the appearance of repulsion of the dispersion curves in the ZGV region. As a result, there exist two solutions, corresponding to two evanescent waves, in which the imaginary parts of the wave number have opposite signs. The size of the appearing gap increases with an increase in the layer conductivity and, at large values, leads to the transformation of an evanescent wave with a positive imaginary part of the wave number (A_1_^e+^) into a forward wave. At the same time, the second evanescent mode with a negative imaginary part of the wave number (A_1_^e−^) is retained.

The similar situation has been demonstrated earlier when the liquid loading on the aluminum plate results in the hybridization of the real and the complex branches with the splitting of the previously continuous curve into two separate complex branches [15]. 

Figure 8 also shows the auxiliary lines Re(*V_ph_*) *= λf* for *λ* = 1.46 mm and *λ* = 1.4 mm. It can be seen that by changing the conductivity of the surface layer, it is possible to achieve the appearance or disappearance of the corresponding acoustic signal in a given frequency range. This is of practical interest in the development of physical sensors and signal processing devices.

**Figure 9:**
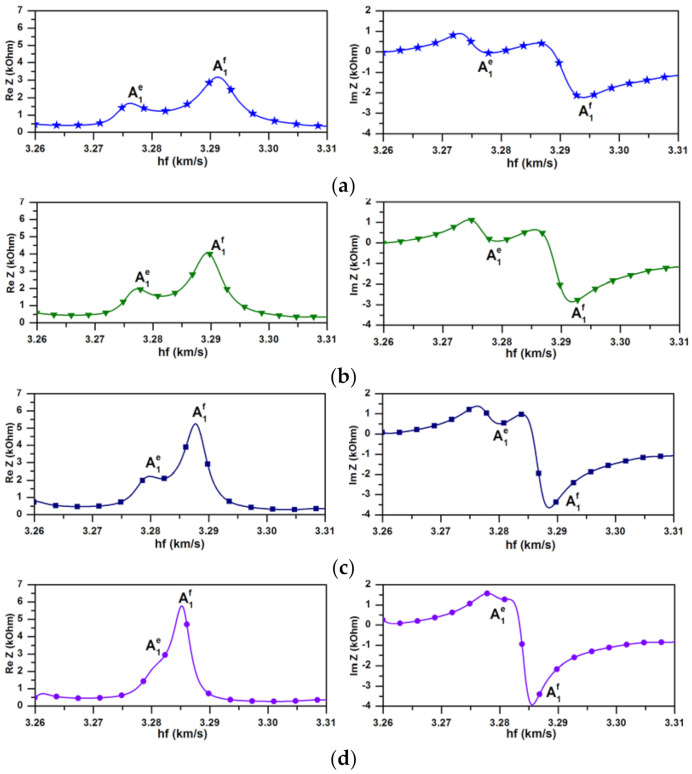
The dependencies of the real Re (left columns) and imaginary Im (right columns) parts of the electrical impedance *Z* of IDTs with the spatial periods λ of (**a**) 1.4 mm, (**b**) 1.42 mm, (**c**) 1.44 mm, (**d**) 1.46 mm on the parameter *hf* obtained by 3D FEM modeling A_1_^e^ and A_1_^f^ show the positions of the peaks of the corresponding evanescent and forward waves, respectively.

The frequency dependencies of the real Re and imaginary Im parts of the electrical impedance Z of the IDTs with spatial periods of 1.4, 1.42, 1.44 and 1.46 mm, obtained as a result of 3D FEM modeling are presented on Figure 9. Here the frequency is normalized by the plate thickness *h* = 0.49 mm. It can be seen that, the resonant frequency of the forward A_1_^f^ wave decreases with increasing period of the IDT. At that the resonant frequency of the evanescent wave A_1_^e^ increases with increasing period of the IDT and the resonances of A_1_^e^ and A_1_^f^ waves merge near the ZGV point. It should also be noted that the value of the electrical impedance corresponding to the evanescent wave A_1_^e^ is much less than that for the forward wave A_1_^f^. This indicates the presence of an additional mechanism of energy dissipation and can serve as evidence of the vanishing nature of this wave.

### 3.2. Experimental Results

Based on the lines presented in Figure 4a and the performed 3D FEM modeling the values of *λ* = 1.4 mm, 1.42 mm, 1.44 mm, and 1.46 mm were chosen for the experimental confirmation of the evanescent wave existence.

**Figure 10:**
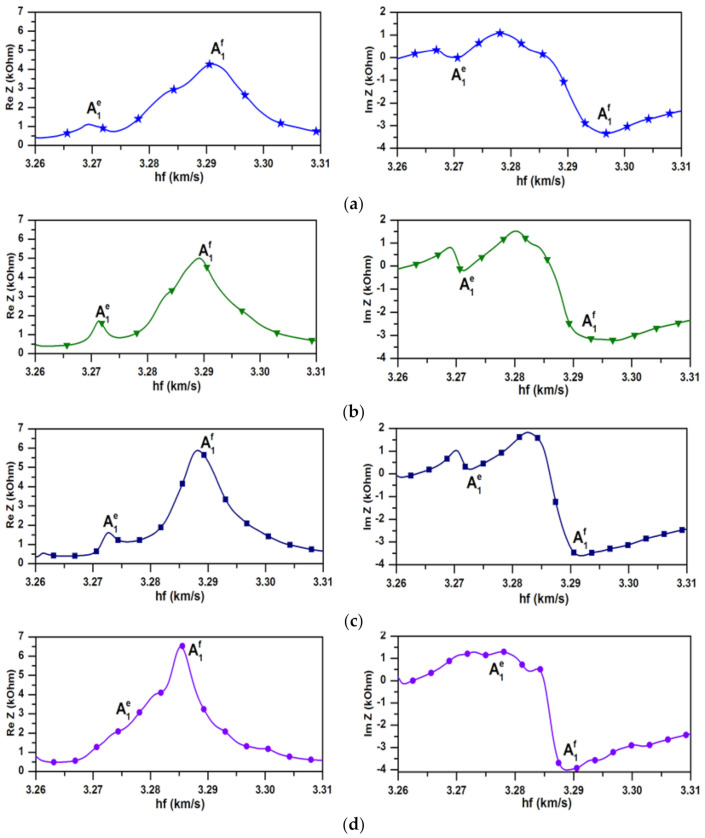
The experimental dependencies of the real Re (left columns) and imaginary Im (right columns) parts of the electrical impedance Z of the IDTs with the spatial periods *λ* of (**a**) 1.4 mm; (**b**) 1.42 mm; (**c**) 1.44 mm; (**d**) 1.46 mm on the parameter *hf.* A_1_^e^ and A_1_^f^ show the positions of the peaks of the corresponding evanescent and forward waves, respectively.

The experimentally measured frequency dependencies of the real Re (a) and imaginary Im (b) parts of the electrical impedance *Z* of the IDTs with the spatial periods *λ* of 1.4, 1.42, 1.44 and 1.46 mm are presented in Figure 10. The variation of the wafer thickness in range of 0.489–0.493 μm was taken into account at normalization.

The theoretical and experimental values of normalized resonant frequencies *hf* of the IDTs with different spatial periods corresponding to A_1_^e^ and A_1_^f^ waves are presented in Table 1.

It can be seen that, as predicted in theory, the resonant frequency of the forward wave A_1_^f^ decreases with an increase in the IDT period. At that the resonant frequency of the evanescent wave A_1_^e^ increases with increasing period of the IDT and the resonances of A_1_^e^ and A_1_^f^ waves merge near the ZGV point. The graphs presented in Figure 9 and Figure 10 show good quantitative and qualitative agreement between the theoretical and experimental results.

The obtained experimental results confirm our earlier assumption [42] about the possibility of excitation and registration of the so-called evanescent waves. As a result of the work performed, it was found that the frequency region of existence of these waves is limited to the region near the ZGV point. Judging by the results obtained, these waves are an integral part of dispersion curves, which have a branch of backward waves.

## 4. Conclusions

A system of the acoustically isolated IDTs with the different spatial period suggested earlier was used for the experimental registration of the evanescent acoustic wave in piezoelectric plate in the first time. The spatial periods of the IDTs corresponding to the dispersion curve of the antisymmetric Lamb wave of the 1st order (A_1_^f^) as well as evanescent acoustic wave (A_1_^e^) near the ZGV point in YX LiNbO_3_ plate were calculated by using 2D ordinary differential equations. The experiments confirmed the existence of the evanescent backward wave in the considered crystallographic orientation. The obtained experimental results were compared with the results of modeling performed by using the 3D FEM commercial software COMSOL 5.3. The experimental results turned out to be in a good agreement with the theoretical ones. Due to proximity of evanescent waves to a ZGV point its properties should be extremely sensitive to the change in the quality of the piezoelectric plate and contacting object. The obtained results have significance for the development of the nondestructive waveguide analysis based on the evanescent waves. The influence of an infinitely thin layer with arbitrary conductivity placed on a plate surface was also investigated. It has been shown that the frequency region of an evanescent acoustic wave existence is very sensitive to the changes in electrical boundary conditions. The performed calculations have shown that an increase in a layer conductance can reduce the attenuation of an evanescent wave down to zero and thereby transform this wave into a propagating one. Thus, it becomes possible to detect a hidden defect in a piezoelectric plate. If a traveling wave propagates in such a plate and interacts with the defect, various types of evanescent waves are generated. If a layer is placed on the surface of the plate under investigation, the conductivity of which can be changed, for example, by an electric field [43,44], then at certain conductivity an additional traveling wave will appear, indicating the presence of a defect. It is obvious that the implementation of this method requires additional calculations and experiments.

## Figures and Tables

**Table 1:** The theoretical and experimental values of normalized resonant frequencies of the isolated interdigital transducers (IDTs) with different spatial period λ.

λ, mm	Theory		Experiment	
	hf(A_1_^f^), km/s	hf(A_1_^e^), km/s	hf(A_1_^f^), km/s	hf(A_1_^e^), km/s
1.40	3.295	3.275	3.293	3.267
1.42	3.290	3.277	3.290	3.272
1.44	3.288	3.279	3.288	3.274
1.46	3.285	3.283	3.285	3.277

## Data Availability

Not applicable.
